# How CAR T Cells Breathe

**DOI:** 10.3390/cells11091454

**Published:** 2022-04-25

**Authors:** Christopher Forcados, Sandy Joaquina, Nicholas Paul Casey, Benjamin Caulier, Sébastien Wälchli

**Affiliations:** 1 Translational Research Unit, Department of Cellular Therapy, Oslo University Hospital, 0379 Oslo, Norway; christopher.forcados@rr-research.no (C.F.); sandy.joaquina@rr-research.no (S.J.); nicholas.casey@rr-research.no (N.P.C.); benjamin.caulier@rr-research.no (B.C.); 2 Center for Cancer Cell Reprogramming (CanCell), Institute for Clinical Medicine, Faculty of Medicine, University of Oslo, 0372 Oslo, Norway; 3 Department of Molecular Cell Biology, Institute for Cancer Research, Oslo University Hospital, 0379 Oslo, Norway

**Keywords:** metabolism, CAR, tonic signaling, T cells, chimeric antigen receptor

## Abstract

The manufacture of efficacious CAR T cells represents a major challenge in cellular therapy. An important aspect of their quality concerns energy production and consumption, known as metabolism. T cells tend to adopt diverse metabolic profiles depending on their differentiation state and their stimulation level. It is therefore expected that the introduction of a synthetic molecule such as CAR, activating endogenous signaling pathways, will affect metabolism. In addition, upon patient treatment, the tumor microenvironment might influence the CAR T cell metabolism by compromising the energy resources. The access to novel technology with higher throughput and reduced cost has led to an increased interest in studying metabolism. Indeed, methods to quantify glycolysis and mitochondrial respiration have been available for decades but were rarely applied in the context of CAR T cell therapy before the release of the Seahorse XF apparatus. The present review will focus on the use of this instrument in the context of studies describing the impact of CAR on T cell metabolism and the strategies to render of CAR T cells more metabolically fit.

## 1. Introduction

T cells modified with a chimeric antigen receptor (CAR) have demonstrated remarkable clinical efficacy in several B-cell malignancies. However, this strategy has shown less promising results against solid tumors, where heterogeneity, an immunosuppressive microenvironment, and low antigen specificities remain major barriers to effective and safe CAR therapy. A solution resides in optimizing the CAR T cell fitness, which involves a proper understanding of their metabolism.

Metabolism plays a pivotal role in many cellular processes through the maintenance of survival, adaptation (fitness), and specialized functions. Different techniques have been developed to study the cellular respiratory profile through the detection and analysis of metabolic markers. In 1960, the Clark electrodes were used to measure the concentration of glucose and oxygen by electrochemistry, using a platinum catalytic surface [1,2,3]. The respiratory function can now be analyzed at high resolution with fluorescence microscopy by measurement of the mitochondrial calcium, mitochondrial membrane potential, pH, and NAD(P)H autofluorescence. Real-time fluorescence resonance energy transfer (FRET) is also used to estimate the mitochondrial or glucose flux in single live cells [4,5]. Other strategies include the tracking of specific metabolic markers by stable isotopes and intra- or extracellular metabolic sensors [6] using flow cytometry and mass cytometry [7,8,9]. Although a variety of assays exist to assess metabolism, they are usually destructive and/or lack real-time measurements. Furthermore, most of these assays lack in throughput capacity. Recent technologies have emerged to address these issues, among them are Seahorse XF by Agilent and O2k by Oroboros. Both allow for the assessment of the mitochondrial respiration of live cells or isolated mitochondria. Their respective advantages and limitations are further discussed by Horan et al. [10]. In the present review, we have focused our attention on articles describing CAR T cell metabolism using Seahorse XF. First used to analyze tumor cells, the technology has become increasingly popular in the context of immune cell studies. The Seahorse XF analyzer allows high throughput and the concomitant assessment of oxidative phosphorylation and glycolysis by studying the oxygen consumption rate (OCR) and the extracellular acidification rate (ECAR), respectively. These two metabolic parameters are of particular relevance to T cell metabolism, as further discussed below. We will explain the basic principles of metabolism and focus on how they affect T cell fate. We will then connect CAR T cell biology with metabolism and provide recent examples of Seahorse use in CAR T cell development by comparing the different protocols, results, and interpretations.

## 2. General Considerations on Metabolism

Metabolism is defined as all chemical reactions that occur within a living organism, of which catabolism is the breakdown of complex macromolecules into smaller molecules to extract energy, and conversely, anabolism is the building of essential molecules through the use of energy [11]. In other words, it consists of all the chemical reactions taking place inside a cell to maintain homeostasis, including glycolysis, the tricarboxylic acid cycle (TCA; also known as the Krebs cycle), oxidative phosphorylation (OXPHOS), amino acid metabolism, fatty acid metabolism, and cholesterol synthesis. These metabolic pathways are enzymatic reactions enabling the production of either essential structural components, such as cholesterol for the plasma membrane, or energy, mostly in the form of ATP. These reactions are highly interdependent and take place mainly in two cellular compartments: the cytosol and the mitochondria [12,13,14].

**Figure 1:**
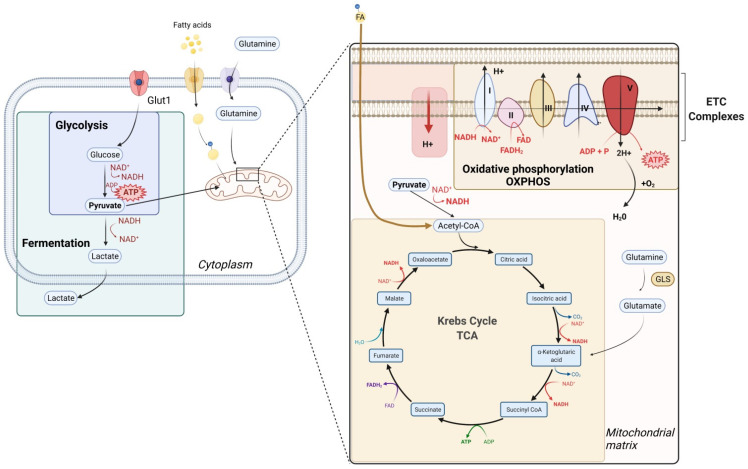
Overview of cell metabolism and Seahorse drugs and associated targets. See text for detailed information. FA = Fatty acids; GLS = Glutaminase.

The dominant energy-yielding catabolic process is a series of oxidation and reduction—termed respiration—resulting in the transfer of electrons from an electron donor towards an electron acceptor (Figure 1, right panel). Two different types of respiration exist—aerobic or anaerobic—which are oxygen-dependent or oxygen-independent, respectively. Aerobic respiration corresponds to a chain of reactions starting with glycolysis, where one glucose molecule is broken down into two pyruvates (Figure 1, left panel). It is followed by pyruvate’s transfer into the mitochondria to enter the TCA after conversion to acetyl-CoA. Lastly, OXPHOS takes place; it is the regeneration of reduced coenzymes (NADH and FADH_2_), generated during the glycolysis or the TCA to their oxidized forms (NAD^+,^ FAD) by the electron transport chain (ETC). The ETC consists of five transmembrane proteins, named complex I to V. Importantly, the fourth complex transfers the electrons to dioxygen (final acceptor), thus creating a differential of electric charges between both sides of the inner mitochondrial membrane. Finally, the fifth complex (also known as ATP synthase) uses this membrane potential to phosphorylate ADP to ATP (Figure 1, right panel) [12].

The whole process is concluded by the release of energy in the form of ATP. Theoretically, 32 ATP molecules will be generated from one glucose molecule. In addition, there is another mechanism quickly generating ATP molecules: fermentation, which bypasses respiration by degrading the pyruvate molecules from glycolysis into lactate [15]. Although an anaerobic process, fermentation is not anaerobic respiration as there is no involvement of an ETC. Importantly, lactate can also be produced in the presence of oxygen, a process known as aerobic glycolysis, or the Warburg effect. This was first described in tumor cells but also recently in lymphocytes [16,17]. Indeed, Otto Warburg and colleagues observed in the 1920s that tumors had a high glucose uptake compared to the surrounding tissue [18]. This led to the hypothesis from Warburg that dysfunctional mitochondria were the root of aerobic glycolysis and that this event was the primary cause of cancer [19,20]. Interestingly, the Warburg effect has also been observed in many cells during rapid proliferation, notably lymphocytes and endothelial cells [21,22,23]. Therefore, aerobic glycolysis likely offers more than simply the provision of large quantities of ATP to highly proliferating cells. A recent hypothesis highlighted the importance of glycolysis in providing carbon to generate biomass and proposed that the major function of aerobic glycolysis is to maintain high levels of glycolytic intermediates, supporting anabolic reactions in cells that are rapidly proliferating [16,20]. Another interesting hypothesis is that aerobic glycolysis not only supports proliferation but also mediates cellular signaling through the binding of the glycolysis enzyme Glyceraldehyde 3-phosphate dehydrogenase (GAPDH) to adenylate-uridylate (AU)-rich elements in the 3′ UTRs of IFN-γ and IL-2 mRNAs, therefore limiting their optimal translation if glycolysis is not engaged [17,21].

Another important metabolic process is fatty acid metabolism which encompasses de novo fatty acid synthesis (FAS) and fatty acid β-oxidation (FAO). FAS is an anabolic process resulting in the synthesis of fatty acids supporting cell proliferation. FAO occurs in mitochondria, in an oxygen-dependent manner where the long-chain fatty acids are degraded to acetyl-CoA. Aside from replenishing the pool of coenzymes and producing ATP, one molecule of palmitate has a total yield of 106 ATP, and the acetyl-CoA acts as a substrate for the acetylation of proteins. Furthermore, acetylation is a major regulator of transcription through histone acetylation and deacetylation, promoting or repressing gene expression, respectively [12]. This process is dependent on glucose-derived acetyl-CoA [24]. Lastly, amino acids can also be catabolized to produce energy; amongst these, we will discuss glutamine as it is also critical for T cell proliferation. In mitochondria, glutaminase (GLS) converts glutamine into glutamate, which is further converted into α-ketoglutarate, thus entering the TCA [25].

All these interlinked processes can be summed up as generating energy (ATP) and the necessary intermediates (through the glycolytic and TCA reactions) for other biosynthetic or downstream pathways. These processes are of utmost importance for cell homeostasis and play a critical role in enabling their specialized functions. For example, activated T cells need more energy and structural components in order to proliferate (e.g., phospholipids and cholesterol) and metabolic intermediates to exert their effector function (e.g., production of signaling proteins and cytokines). Finally, noted but not detailed here, activated T cells also need amino acids, which must be metabolized intracellularly or imported from the surrounding microenvironment [26,27,28].

## 3. T Cell Metabolism

T cells can be classified based on their differentiation state into naïve T (T_N_), T-stem cell memory (T_SCM_), T-central memory (T_CM_), T-terminal memory (T_TM_), T-effector memory (T_EM_), and T-terminal effector (T_TE_) subsets. Different subsets rely upon distinct metabolic pathways [29,30,31].

**Figure 2:**
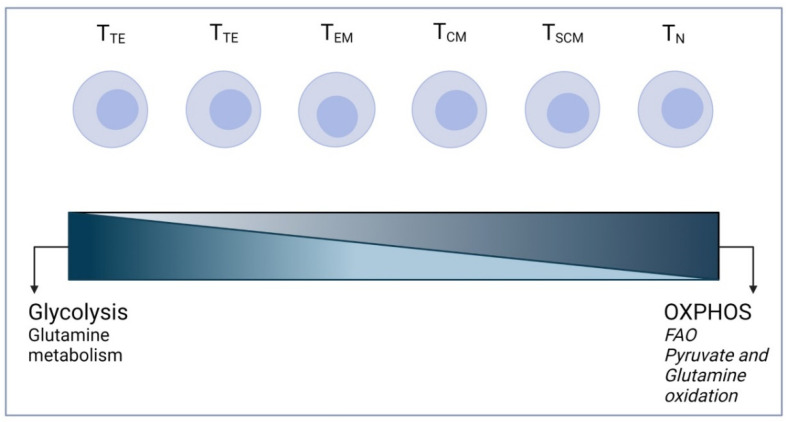
T cells and their metabolic profile. Metabolic reliance of T cells based on their differentiation stage: from right, quiescent T cell, to left, terminally differentiated T cells.

Mature naïve T cells continuously migrate through secondary lymphoid tissues, performing immune surveillance prior to activation. Therefore, they constantly rearrange their cytoskeletons. Although this process is energy-consuming, it requires only basal replacement biosynthesis; thus, their metabolic balance favors energy production over biosynthesis. They mainly rely on FAO, pyruvate, and glutamine oxidation, the three highest energy-yielding processes, through the TCA cycle (Figure 2). Even when they are resting, they need cell-extrinsic signaling to maintain this basal metabolism. This signaling is mediated by the IL-7 receptor and the TCR, both crucial to maintaining cell homeostasis, by regulating, respectively, the cell surface trafficking or expression of Glut1 (the plasma membrane glucose transporter), thus maintaining basal glycolysis [32,33].

T cell activation is orchestrated by TCR/peptide–MHC interaction, providing the first signal (signal 1) and forming an immune synapse. The output of signal 1 is the phosphorylation of the CD3ζ’s immunoreceptor tyrosine-based activation motifs (ITAMs). Further interaction at the synapse with co-stimulatory molecules (e.g., CD28, ICAMs) provides a second signal (termed signal 2). Thus, a complete TCR-based activation of T cells requires these two signals. The foremost metabolic alteration of the naïve T cells (T_N_) upon activation is an increase in glucose uptake and the use of aerobic glycolysis—the Warburg effect (Figure 2). There is also a rise in glutamine oxidation and a decrease in lipid oxidation (FAO). Interestingly, in the context of physiological T cells, CD28 signaling is the primary regulator of Glut1 expression, through the PI3K-AKT signaling axis, during CD3/CD28 stimulation. This leads to increased Glut1 expression and glucose uptake, resulting in glycolysis exceeding the basal needs of the cell for macromolecular synthesis or the maintenance of ATP/ADP levels [34,35].

CD4+ T cells can be subdivided into T helper 1 (Th1), Th2, and Th17—which are all effector CD4+ T cells (Teffs)—and CD4+ regulatory T cells (Tregs). Although other subsets exist, they are less well-defined at the metabolic level. Their metabolic states differ between an emphasis on aerobic glycolysis and decreased lipid oxidation for the Teffs to an emphasis on lipid oxidation and decreased glycolysis for the Tregs. Interestingly, although Tregs glycolysis is decreased compared to that of the Teffs, they possess a higher glycolytic rate than naïve T cells. Their differing metabolic reliance is regulated by different post-transcriptional regulators. In particular, the mammalian target of rapamycin complex 1 (mTORC1) is needed for all CD4+ T cell effector lineage differentiation (Th1, Th2, and Th17) [36]. Among the CD4+ Teff subset, the Th17 cells need glutaminolysis for functional differentiation and have higher rates of glutaminolysis [37,38]. Conversely, glycolysis contributes to Th1 and Th2 differentiation, while FAO—required by Tregs—suppresses CD4+ Teff differentiation [39,40].

CD8+ T cells also undergo a switch from oxidative metabolism to glycolysis upon activation (Figure 2). This transition has been shown to be essential in supporting the differentiation into cytotoxic T cells (CTLs), the division every six to eight hours, and the production of inflammatory cytokines and cytolytic granules (perforin and granzyme B). Once the pathogen is cleared, the majority of the CTLs undergo apoptosis, while a small percentage undergoes further differentiation into long-lasting, quiescent memory CD8+ T cells. These memory CD8+ T cells require basal energy generation to support basic cellular functions and prevent cell death. Compared to CTLs, memory CD8+ T cells express high levels of Carnitine Palmitoyl Transferase I (CPT1a), a mitochondrial lipid transporter, the inhibition of which impedes memory T cell survival. Memory CD8+ T cells also possess an increased ‘spare respiratory capacity’, the excess capacity of mitochondria to induce respiratory metabolism during metabolic stress [33,41].

Additionally, T cells have specific requirements in terms of amino acid availability. Abundance or depletion of key amino acids in the environment will directly or indirectly impact T cell physiology. Notably, leucine and glutamine are critical for optimal activation and proliferation and are also involved in CD4+ T cell differentiation, while arginine supports survival. Furthermore, serine intake or synthesis has direct implications for the effector functions [41].

Taken together, the effector T cells have a distinct metabolic signature, favoring glycolysis over OXPHOS (aerobic glycolysis, Warburg effect) and decreasing lipid oxidation. Yet, upon antigen clearance, effector T cells do not persist. Conversely, memory T cells have a decreased glycolytic rate—although it is still higher than that of naïve T cells—and rely mostly upon lipid oxidation. T cell-based cellular therapy depends on the capacity of the therapeutic product to persist and engage in their tumor-targeted cytotoxicity.

## 4. Importance of Studying CAR T Cell Metabolism

CAR T cell therapy aims to specifically eliminate tumor cells in a sustained manner. Aside from the undesirable toxicities and the on-target off-tumor-related toxicity, CAR T cell therapies also face several disease-related challenges. Among these, the most common is the strong tumor microenvironment (TME), which encompasses different effects, such as antigen loss and immunosuppression, both resulting in serious, deleterious consequences for the therapeutic immune product. In addition, the TME features conditions unfavorable for CAR T cell survival, such as poor nutrient availability, the presence of immunosuppressor cells such as Tregs and myeloid-derived suppressor cells (MDSCs) downregulating the T cell effector functions, and localized hypoxia. Furthermore, the dense arrangement of the cells and the extracellular matrix presents a physical barrier for immune cell infiltration and function [42]. Hypoxia and nutrient scarcity (e.g., of glucose and amino acids) are major impediments to the T cell metabolic functions, precluding full exertion of the immune functions [43,44].

CARs are synthetic receptors which redirect T cells against a defined target, while co-opting the TCR signaling machinery. Therefore, second generation CARs—the common clinical format [45]—were designed to bear a CD3ζ domain, mimicking signal 1, and a co-stimulatory domain, providing signal 2. The co-stimulatory molecules used in CARs vary but are typically of the tumor necrosis factor receptor superfamily (e.g., 4-1BB/CD137, OX40/CpavoD134) or the immunoglobulin superfamily (e.g., CD28, ICOS/CD278) [46]. Notably, in the physiological T cell context, CD28 signaling is the primary regulator of Glut1. This leads to glucose uptake, resulting in the glycolysis exceeding the basal cellular requirements for macromolecular synthesis or the maintenance of the ATP/ADP pool [34]. Although less frequently investigated, ICOS of the CD28 family receptors has been described as enhancing glucose uptake and metabolism via mTOR activation (in follicular helper T cells, Tfh), as well as favoring Th17/Th1 T cell differentiation when used in a CAR design [47,48]. Similarly, 4-1BB (CD137) of the TNFR family promotes glycolytic metabolism upon activation through Glut1—though to a lesser extent than CD28—and promotes a central memory phenotype in T cells (whereas CD28 promotes an effector memory phenotype in the CAR T cell setting) [49]. Finally, CD137 has been shown to enhance FAO [50]. Concerning OX40, another member of the TNFR family, there is so far no concrete experimental evidence of the biology of its activation [51].

**Figure 3:**
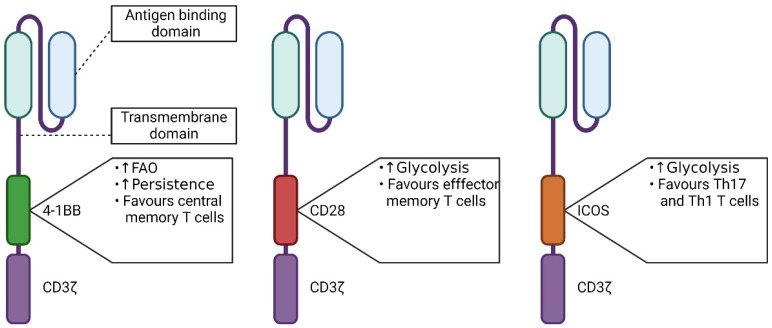
Schematic representation of CAR molecules of the second generation and the metabolic consequences of the different co-stimulatory domains. From left to right: 4-1BB (CD137); CD28; and ICOS co-stimulatory domains.

CARs directed against the same target but harboring different designs could promote or impair a given metabolic pathway over another, leading to either an inefficient activation or an unwanted lineage commitment, as shown with CARs constructed with either CD28 or 4-1BB co-stimulatory domains (Figure 3) [49]. The investigators compared CD19-targeting CARs, bearing either a 4-1BB or a CD28 co-stimulatory domain, for T_CM_ marker (CD45RO, CCR7) expression or for metabolic signature (OXPHOS, glycolysis, and fatty acid metabolism). They showed that the co-stimulatory domain used in the CAR design induces distinct metabolic profiles and subset fate [49]. Similarly, W. Li et al. [52] and Liu et al. [53] demonstrated that either preventing ubiquitination of the CAR or polarizing the CAR T cells during production induces distinct metabolic profiles and T cell fates, thus improving their function in vivo. In addition to the study of CAR T cell subsets, the investigation of basal and activated metabolic aptitudes can help in the gaining of insights that are valuable for decision making throughout the investigation of a CAR candidate.

Energetic metabolism is intrinsically tied to the mitochondria. Their role in ATP production is of paramount importance for all cells. The investigation of T cell function and differentiation revealed an intimate interplay between their metabolic profile and their subset fate, as briefly discussed above. As such, along with the influence of CAR design and the co-stimulation domains used, the direct reprogramming of mitochondrial metabolic pathways is emerging as a strategy to develop long-lasting and functional CAR T cells [54,55,56]. Indeed, as demonstrated in the studies cited herein, the alteration of mitochondrial function is correlated with the alteration of T cell fate and thus the function and potency for persistence. Therefore, hindering or favoring mitochondrial metabolic pathways can shift the balance of T cell differentiation, opening new avenues for the optimization of CAR T cell end products [57,58].

## 5. Study of Metabolism in CAR T Cells

**Figure 4:**
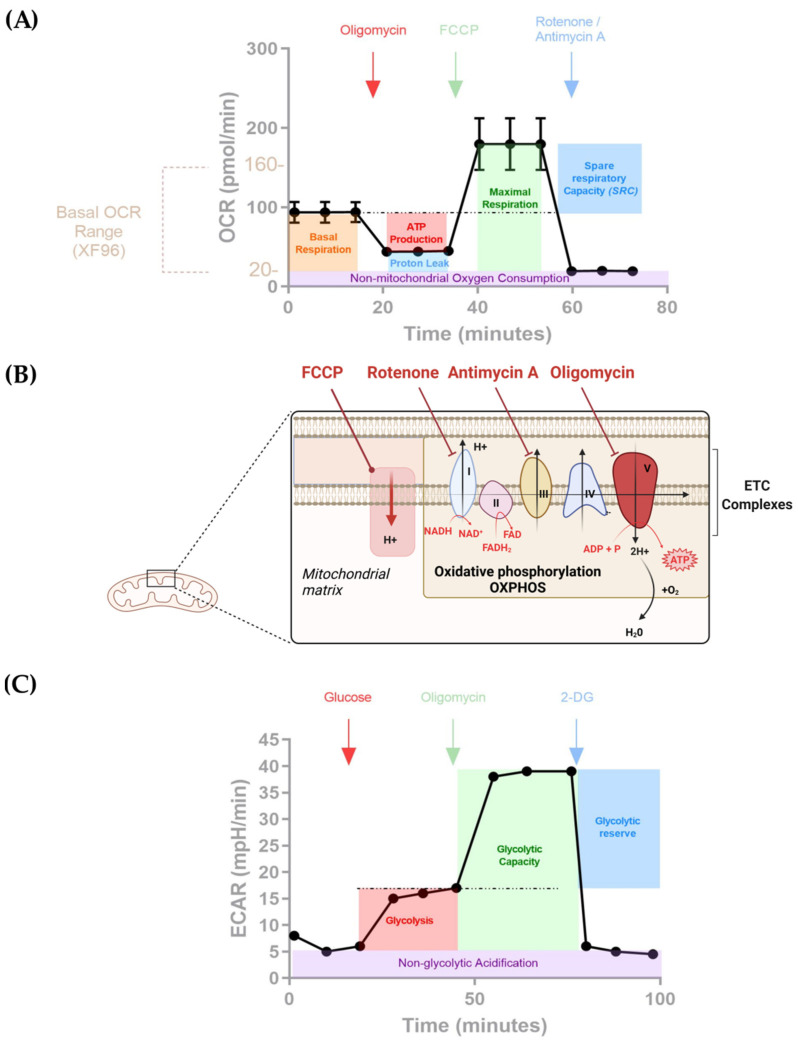
(**A**) Seahorse XF Cell Mito Stress Kit. The mitochondrial bioenergetics function is assessed through the measurement of the Oxygen Consumption Rate (OCR). First, a basal OCR value is recorded in triplicate, which reflects mitochondrial activity at a steady state. Then, drugs are sequentially used to challenge components of the mitochondrial respiration chain along OCR measurement 1 = Oligo ATP synthase inhibitor, 2 = FCCP uncoupler of mitochondrial oxidative phosphorylation, and 3 = Rotenone/Antimycin A, inhibitors, respectively, of complex I and III of the ETC. The measurements are repeated 3 times. (**B**) Overview of the ETC and the targets of the drugs used in the XF Cell Mito stress kit. (**C**) Seahorse XF Glycolysis stress test kit. The glycolytic function is assessed through the measurement of Extracellular Acidification Rate (ECAR). First, cells are incubated in stress test medium without glucose or pyruvate and the ECAR is measured. Then, the first injection is a saturating concentration of glucose; measurements taken during that time indicate glycolysis under basal conditions. The second injection of Oligomycin, an ATP synthase inhibitor, permits, through the measurement of ECAR, assessment of the maximum glycolytic capacity. Lastly, 2-deoxy-glucose (2-DG) is injected. This is a glucose analog that binds competitively to glucose hexokinase, the first enzyme in the glycolytic pathway. The resulting decrease proves that the ECAR previously measured is due to glycolysis.

It is therefore important to benchmark a CAR T cell metabolic assessment method by harmonizing the protocols in order to capture the impact of metabolism for future CAR T cell therapies. The studies involving Seahorse analyzers and CAR T cells were mainly performed using the Mito Stress Kit, whereby mitochondrial respiration is challenged with OXPHOS inhibitors (Table 1 and Figure 1). Briefly, specific agonists or antagonists of the ETC are sequentially administered throughout the experiment, and the variations in OCR and ECAR are measured. The measurements are operated using fluorescent sensors fitted in a bio-cartridge [59]. First, oligomycin, an inhibitor of ATP synthase [60], decreases mitochondrial respiration and therefore oxygen consumption. Second, carbonyl cyanide-p-trifluoromethoxyphenylhydrazone (FCCP) uncouples the mitochondrial proton gradient necessary for ATP production, which induces maximum oxygen consumption through complex IV. Finally, Rotenone and Antimycin A, which are, respectively, complex I and III inhibitors, are injected, inducing a mitochondrial respiration blockade (Figure 1) [61,62,63]. Altogether, the drug-induced variations inform on the key mitochondrial parameters through OCR variations (such as basal respiration, ATP-synthesis linked respiration, and proton leak, which can inform on mitochondria damage or be used as a mechanism to regulate mitochondrial ATP production) and the spare respiratory capacity (SRC) (Figure 4A). The latter is of great importance as it characterizes the cell’s ability to meet an energetic challenge and therefore reflects specific T cell functions, such as activation, proliferation, or differentiation. In addition, ECAR measures the rate of extracellular acidification, which mainly comes from the accumulation of lactic acid in the medium (glycolytic pathway) [64]. Among the studies reported, some used the Seahorse technology to explore the influence of CAR signaling tails (4-1BB vs. CD28) [49,65], while others focused on comparing CARs with either different designs or targets [52,66,67,68,69], the additional secretory functions [53,70], the combination with PD-1/PD-L1 blockage [71], the polarization of the T cells [53,72], or the influence of co-expressing enzymes alongside the CARs in T cells [73,74].

### 5.1. The Influence of the Signaling Domain

The articles comparing the CD28 and 4-1BB co-stimulatory domains used either a mesothelin CAR or a CD19 CAR [49,65]. They observed differences between 4-1BB CARs and CD28 CARs in terms of basal OCR and SRC. In the study of Kawalekar et al. [49], CARs with a 4-1BB co-stimulatory domain showed a higher basal OCR and SRC than the CD28 CAR. Interestingly, extensive investigation of the candidate genes of the oxidative or glycolytic pathways showed a differential expression between the 4-1BB and CD28 CAR T cells that was consistent with the Seahorse assay results. Similarly, the ratio of mitochondrial mass to cell mass and the expression levels of the mitochondrial genes (encoded by either the nuclear or the mitochondrial genome) were increased in CAR T cells with 4-1BB co-stimulation. The observed difference in SRC strongly hints at a reprogramming of the transcriptional networks by the 4-1BB CAR, leading to enhanced mitochondrial biogenesis and oxidative metabolism. The authors showed that the co-stimulation domain used in the CAR design strongly impacts the metabolic profiles and lineage fate of the T cells. While Liu and colleagues [65] observed a lower basal OCR with 4-1BB CARs than with CD28 CARs, the SRC was also higher with 4-1BB CARs. In their study, CARs bearing the two different co-stimulatory domains were used to validate and model exhausted tumor-infiltrating lymphocytes (TILs) in human hepatocellular carcinoma (HCC). Notably, to gain insights into the mechanism leading to these metabolic differences, the authors measured the expression of candidate genes but focused on glycolytic and lipid metabolism. They showed that adding a 4-1BB signaling domain to the CAR makes the CAR T cells downregulate the expression of several genes involved in glycolysis and upregulate the genes associated with mitochondrial FAO. They also confirmed that the surface expression of CD137 is a marker of exhausted T cells (Tex) with superior effector functions and proliferation potential. Moreover, CD137+ Tex had a higher fatty acid-binding protein 5 (FABP5) expression. Inhibiting the FABP5 expression and the mitochondrial FAO impaired the anti-apoptotic and proliferative capacities of CD137-enriched Tex [65]. Both teams demonstrated that CAR T cells possessing the 4-1BB domain were more likely to use the OXPHOS pathway, which characterizes memory T cells, thus supporting central memory differentiation and T cell persistence. In both studies, the experimental conditions were different with respect to the number of cells and the concentrations of the drugs used (Table 1). In fact, Kawalekar et al. [49] used both Seahorse XF24 and XF96 with 1 × 10^6^ cells/well, while Liu et al. [65] used 1.5 × 10^5^ cells/well. Agilent advises a cell monolayer in order to avoid hypoxia, which could affect the metabolism of the cells. As such, 1 × 10^6^ T cells in a 96-well plate might be too dense, suggesting that the results obtained might be influenced by stressful culture conditions. In addition, the second study also used Etomoxir, which is a fatty acid oxidation inhibitor (FAO) [50,75], without further explanation. It should also be noted that these studies did not show results from untransduced (mock) controls (T cells without CARs), which would have provided an overview of the basal metabolic conditions of the T cells. This would have strengthened the conclusions made.

### 5.2. The Influence of the CAR Design

The pioneer work of W. Li et al. [52] showed that the blocking of CAR downmodulation by inhibiting its ubiquitination affected lysosomal degradation while promoting the recycling of internalized CARs to the cell surface. Inhibiting CAR ubiquitination also led to enhanced endosomal signaling promoting oxidative phosphorylation and memory T cell differentiation, leading to better in vivo persistence. Importantly, the authors based their initial conclusions on observations of a higher OCR and SRC for ubiquitination-deficient CD19 CAR T cells, which they further confirmed with additional techniques. The second study was focused on the tonic signaling of CARs and, in particular, Treg CARs [68]. The authors observed an exhaustion profile for Tregs expressing CARs prone to tonic signaling. Although stable and suppressive in vitro, the cells failed to maintain in vivo function in a xenogeneic model of graft-versus-host disease (GvHD). Indeed, Lamarche and colleagues [68] obtained a higher OCR and ECAR, and a lower SRC, for tonic-signaling CARs, which led them to the conclusion that tonic CAR T cells preferentially exploited a glycolytic pathway. Importantly, these results complemented transcriptome analysis showing changes in metabolic pathways and a phenotypic analysis demonstrating an exhaustion profile. Moreover, they were obtained using different T cells donors, strengthening their conclusions. Lastly, Hirabayashi et al. [69] compared a dual CAR approach with a classical CD28 second-generation CAR. Their dual CAR consisted of a polycistronic vector expressing both the second generation GD2 CAR (GD2.28ζ) and a B7-H3-4-1BB (B7-H3.BB) receptor. This CAR design allows for rapid antitumor effects in vivo, protects from tumor re-challenge, and prevents tumor escape resulting from low antigen density. Moreover, it retains both the functions of the induction of glycolysis and the preservation of OXPHOS. They used an XF24 apparatus with both the Cell Mito stress and the Cell Glycolytic stress kits to assess the metabolic profile of CAR T cells and compared the values obtained at days 0, 1, and 5 post-CAR activation, using 5 × 10^5^ cells in a 24-well plate. The GD2.28ζ/B7-H3.BB dual CAR demonstrated higher glycolytic and OXPHOS activity than GD2.28ζ. Their assay was strengthened by the use of three independent experiments.

### 5.3. Polarization of CAR T Cells

The metabolic changes associated with CAR T cell polarization were also the focus of several recent studies. For instance, Liu et al. [53] examined the metabolism of Th1- and Th9-polarized T cells using Seahorse XF technology and showed that Th9 featured higher OCR and SRC than Th1. Furthermore, Th9-polarized CAR T cells secreted IL-9, expressed lower levels of exhaustion markers, maintained strong proliferative capacities, and had a central memory phenotype and a greater antitumor activity in vivo than Th1-polarized CAR T cells. These findings may not only broaden our understanding of T cell polarization at the metabolic level but may also open the way for the production of a CAR T cell product adapted to solid tumors. However, it should be noted that the basal measurement of the OCR value was outside the range defined by Agilent for the XF24 instrument (600 pmol/min for 50–400 pmol/min). On the same line, Sabatino et al. [72] focused on characterizing CD19 CAR in CD8+ T cells enriched in the T_SCM_ subset. These CD19 CAR CD8+ T_SCM_ cells exhibited an enhanced metabolic fitness and had a robust and long-lasting antitumor effect in an acute lymphoblastic leukemia xenograft model. They observed across five different donors that CD19 CAR T_SCM_ cells displayed a higher SRC than CD19 CAR T cells without T_SCM_ enrichment. As previously mentioned (Figure 2), memory T cells favor OXPHOS while effector T cells favor glycolysis; therefore, memory T cells have a higher SRC compared to effector T cells. Thus, this study highlights the importance of assessing the metabolism of the CAR product in order to select an appropriate T cell population to further improve persistence and achieve robust antitumor responses.

### 5.4. Effect of the Combination of CAR T Cells and Combinatorial Designs

A set of studies also examined the T cell metabolic outcome following various stimulations. Serganova et al. [71] compared naïve, PHA-stimulated, and anti-human prostate-specific membrane antigen (PSMA) CAR T cells, while looking at the kinetics of OCR and ECAR up to 15 days after T cell isolation. The aim of their study was to assess the combination of CAR targeting PSMA with a PD-1/PD-L1 blockade. They observed that the PD-1/PD-L1 blockade resulted in a short-term enhanced response, suggesting other immunomodulatory mechanisms restricting CAR T cells in their prostate cancer model. The authors observed that naïve T cells featured slightly increased basal OCR and ECAR at day 2, which declined over time. Conversely, CAR T cells showed only a modest increase in basal and maximal OCR, starting from day 8, and remained constant until day 15. Concerning basal ECAR variation, the same observation was made following PHA treatment, suggesting that unspecific and specific stimulation yielded similar levels of ECAR. These data can be considered strong because they were the means of values obtained from different donors. In addition to the Mito Stress Kit, these last two studies [71,72] also used the Glycolysis Stress Test Kit (Figure 4C). Apart from studying the CAR influence on the T cell phenotype, other researchers have focused upon capturing the direct metabolic impact of introducing a modified/enhanced CAR construct in comparison to a conventional one. In the work of Zhang et al. [70], CD19 CAR T cells co-expressing or not a soluble PD-1 receptor (sPD-1) were analyzed. The co-expressing T cells led to a reduced tumor burden and prolonged survival in an in vivo tumor model of high PD-L1 expression, compared to conventional second-generation CAR T cells. Here, the authors obtained similar levels of OCR in all conditions (mock, CD19 CAR, and CD19 CAR-sPD-1)—with or without a 48 h co-culture with target tumor cells—and observed that the OCR of the CD19 CAR-sPD-1 T cells was slightly higher, and the ECAR lower, compared to the CD19 CAR T cells. In conclusion, the CD19 CAR-sPD-1 T cells showed similar levels of OXPHOS, but reduced glycolysis, compared to the CD19 CAR T cells, regardless of the presence of tumor cells. Here again, the authors used different complementary experiments to draw conclusions on the metabolism of CAR T cells. In particular, they performed RNA sequencing and showed that their CD19-sPD1 CAR T cells were less differentiated and less exhausted and activated less glycolytic pathway-related genes. We have recently presented an approach to improving the efficacy of CD19 CAR T cells by combining CD19 CAR with an anti-IgKappa (IGK) CAR, called Kz19BB [66]. With this combinatorial CAR design, we showed that we could keep the specificity of IGK CAR while attenuating its serum sensitivity. We studied the metabolism of the T cells expressing different CARs and observed a similar metabolic profile for the anti-IgK CAR and the Kz-19BB CAR when stimulated with a specific antigen (kappa light chain). An inverted construct, 19z-KBB CAR, showed a similar metabolic profile to the CD19 CAR, demonstrating that the metabolic changes observed were solely due to the CD3z-containing construct. These results were obtained with one donor and corroborate additional in vitro results. Elsewhere, another group has tested a different combination of targeting units against GD2 and B7H3 antigens. There, the authors used the synthetic Notch system (CAR GD2-B7H3) [67] to promote resistance to exhaustion and improve the metabolic fitness of the expressing cells. Briefly, this strategy was based on the transcriptional control of the B7-H3 CAR by a GD2 SynNotch CAR. This SynNotch-gated CAR T cell controlled the tumor burden both in vitro and in vivo. Moreover, the fatal neurotoxicity reported using the GD2 CAR T cell was linked to the use of the CD28 co-stimulation domain as this was not observed when a 4-1BB co-stimulation domain was used. With this arrangement, GD2-B7H3 T cells showed a similar rate of oxygen consumption to that of the non-transduced cells, and a higher SRC compared to conventional B7H3 CAR after 48 h of co-culture with target cells. This indicated a greater capacity for oxidative metabolism. This was confirmed by Gene Set Enrichment Analysis, which demonstrated a significant increase in the transcription of glycolytic exhaustion genes in conventional CAR-T B7H3 cells compared to GD2-B7H3 cells.

### 5.5. CAR Modified to Enhance T Cell Resistance to TME

Finally, two recent studies [73,74] evaluated the impact of the co-expression of enzymes affecting metabolism, alongside CARs, in order to generate constructs equipped to fight a strong TME. The enzymes used, *Lactobacillus brevis* NADH Oxidase (*Lb*NOX) [73] and D-2-hydroxyglutarate dehydrogenase (D2HGDH) [74], were expected to provide resistance to the TME by affecting mitochondrial respiration. Co-expression of *Lb*NOX with a mesothelin-specific CD28ζ CAR (CAR_Nox) induced higher levels of baseline OCR compared to a CD28ζ CAR co-expressed with GFP (CAR_GFP), reaching levels similar to those of untransduced T cells. The addition of lactate increased the OCR levels for the CAR_Nox. Subsequent treatment with Rotenone and Antimycin A did not impair the CAR_Nox T cells OCR as much as the OCR of untransduced and CAR_GFP T cells, validating the *Lb*NOX activity in vitro. In addition to the inclusion of an untransduced T cell control, this study specified the use of three donors, strengthening the validity of the observations. Therefore, co-expressing LbNox with the CAR in T cells resulted in enhanced oxygen and lactate consumption, as well as increased pyruvate production and resiliency to lactate dehydrogenase inhibition but did not confer an increased antitumor efficiency in vivo. The second study was more promising; Yang et al. [74] tested CD19 CAR T cells with a *D2HGDH* knocked-out (KO) or overexpressed (OE) backgrounds. D2HGDH is a mitochondrial protein shown to catabolize a component present at high levels in the TME, D-2-hydroxyglutarate (D2HG). In the D2HGDH-OE background, CAR-T cells showed a significant decrease in basal and maximal OCR, which corroborates their flow cytometric observation of an expanded effector memory subset in D2HGDH-OE CAR-T cells. This was further complemented by an in vivo assessment of D2HGDH-OE CAR-T cells, which showed increased tumor control and persistence. Accordingly, the opposite was observed in the KO background. Thus, affecting metabolism seems to represent a reliable route to resisting the TME.

Together, these studies used the Seahorse technology to investigate the differentiation and stimulation states of CAR T cells. Overall, when higher OCR rates were detected, it reflected the involvement of OXPHOS, which is associated with a memory T cell phenotype and therefore a more persistent state. In contrast, a high ECAR associated with a preference toward glycolysis and was associated with an effector T cell phenotype. We noticed that the ECAR value was not taken into account in all the studies (including our own). The ECAR values complement the OCR values, allowing an overview of both the OXPHOS and the glycolytic pathways. Another key point is the influence of the co-stimulatory domain on the differentiation state of transduced T cells. Notably, a CD28 co-stimulatory domain favors an effector memory phenotype, while 4-1BB favors a central memory phenotype. On a technical note, we noticed that the majority of the articles did not indicate the number of technical replicates (Agilent recommends doing six) and the number of biological replicates (or the number of donors used). These are important aspects of the analysis since variation between individuals can sometimes be greater than the effect of the drug in one individual. Furthermore, non-transduced T cells were not always used as a control (Table 1). As such, intra- and inter-study comparisons were difficult to capture; indeed, some baselines were drawn using mock T cells while others were made with irrelevant CAR-expressing T cells. Finally, we also detected that cell density and drug concentrations were not homogeneous between the studies and have combined this review with a technical report (Joaquina et al., manuscript co-submitted) that could be used as basis to establish a unified CAR T cell metabolism analysis using Seahorse.

## 6. Conclusions

Although the metabolic state of CAR T cells is highly relevant to the understanding of CAR-T cell biology, its importance in predicting the clinical efficacy of a CAR construct is not yet clear, and its manipulation may not lead to improved therapeutic outcomes. Despite that, across the several studies that used complementary methods, CAR T cells with an increased SRC correlated strongly with a memory T cell phenotype. It is thus a valuable tool to compare several CAR designs and draw conclusions regarding the broad phenotypes they tend towards. Moreover, this reprogramming has been shown to be modulated mostly by the co-stimulatory domains used in the CAR design. On its own, the assessment of metabolism provides valuable data with which to build a prediction matrix for future clinical trials.

## Figures and Tables

**Table 1:** Selected publications using the Seahorse XF analyzer to study CAR T cell metabolism. CAR studies involving hematopoietic and solid tumor are depicted in light and dark orange, respectively.

CAR	Seahorse					Reference
	Seahorse XF	Kit	Drugs	Number of Cells/Wells	Cell Stimulation	
CD19 CAR with a CD28 co-stimulatory domain/CD19 CAR with a 4-1BB co-stimulatory domain and Mesothelin CD28-CAR/Mesothelin 4-1BB CAR	Seahorse XF24 and 96	Cell Mito Stress kit	Oligomycin/FCCP/Rotenone Antimycin A	1 × 10^6^	Before/After anti-idiotype stimulation (for 7 days and 21 days)	[49]
CD19 CAR with or without T_SCM_-enrichment	Seahorse XF24	Cell Mito Stress kit - Glycolysis Stress kit	Oligomycin/FCCP/Rotenone Antimycin A - Oligo/Glucose/2DG	1 × 10^6^	Not specified	[72]
Anti-hPSMA CAR/Untransduced T cells	Seahorse XF96	Cell Mito Stress kit - Glycolysis Stress kit	Oligomycin/FCCP/Rotenone Antimycin A - Oligo/Glucose/2DG	3.5 × 10^5^	Phytohaemagglutinin (PHA) Stimulation	[71]
Mesothelin CD28-CAR/Mesothelin 4-1BB CAR identification	Seahorse XF96	Cell Mito Stress kit	Oligomycin/FCCP/Rotenone Antimycin A + Etomixir	1.5 × 10^5^	Not specified	[65]
CD19 CAR/CD19 CAR with ubiquitination blocked by mutating all lysines in the CAR cytoplasmic domain	Seahorse XF24	Cell Mito Stress kit	Oligomycin/FCCP/Rotenone Antimycin A	1.5 × 10^5^	After stimulation with irradiated target cells for 14 days	[52]
CD19 CAR/CD19 CAR releasing human soluble PD-1 protein (sPD-1 CAR T) /Untransduced T cells	Seahorse XF96	Cell Mito Stress kit	Oligomycin/FCCP/Rotenone Antimycin A	Not specified	Before/After 48h in co-culture with target tumor cells	[70]
GD2 CAR/CD19 CAR/Untransduced T cells	Seahorse XF96	Cell Mito Stress kit	Oligomycin/FCCP/Rotenone Antimycin A	2 × 10^5^	Not specified	[68]
IGK CAR/IGK CD-19 CAR	Seahorse XF96	Cell Mito Stress kit	Oligomycin/FCCP/Rotenone Antimycin A Anti A	1 × 10^5^	After stimulation with surface-coated specific (IgG) and unspecific (anti-CD3) antibodies	[66]
GD2 CAR/GD2-B7H3 CAR /Untransduced T cells	Seahorse XF24	Cell Mito Stress kit	Oligomycin/FCCP/Rotenone Antimycin A	1 × 10^6^	After 48h in coculture with target tumor cells	[67]
CD19 CAR Th9 or Th1 polarization	Seahorse XF24	Cell Mito Stress kit	Oligomycin/FCCP/Rotenone Antimycin A	Not specified	Not specified	[53]
Mesothelin CD28 CAR with *Lb*NOX or GFP	Seahorse XF96	Cell Mito Stress Kit	Sodium–L–lactate/Rotenone Antimycin A	2 × 10^5^	None	[73]
GD2 CAR with a CD28 co-stimulatory domain/GD2 CAR co-expressed with B7H3 with a 4-1BB co-stimulatory domain	Seahorse XF24	Cell Mito Stress kit - Glycolysis Stress kit	Oligomycin/FCCP/Rotenone Antimycin A - Rotenone/Antimycin A/2DG	5 × 10^5^	After stimulation with surface-coated specific immunoglobulin (1A7 mAb) and chimera (4Ig-B7-H3) for CAR activation	[69]
CD19 CAR with a 4-1BB co-stimulatory domain in D2HGDH knocked out or overexpressing T cells	Seahorse XF24	Cell Mito Stress kit	Oligomycin/FCCP/Rotenone Antimycin A	1 × 10^6^	None	[74]

## Data Availability

Not applicable.

## References

[1] Clark L.C., Wolf R., Granger D., Taylor Z. (1953). Continuous Recording of Blood Oxygen Tensions by Polarography. J. Appl. Physiol..

[2] Wang J. (2008). Electrochemical Glucose Biosensors. Chem. Rev..

[3] Clark L.C., Lyons C. (1962). Electrode Systems for Continuous Monitoring in Cardiovascular Surgery. Ann. N. Y. Acad. Sci..

[4] Bittner C., Loaiza A., Ruminot I., Larenas V., Sotelo-Hitschfe T., Gutiérrez R., Córdova A., Valdebenito R., Frommer W., Barros L.F. (2010). High Resolution Measurement of the Glycolytic Rate. Front. Neuroenergetics.

[5] Takanaga H., Chaudhuri B., Frommer W.B. (2008). GLUT1 and GLUT9 as the Major Contributors to Glucose Influx in HEPG2 Cells Identified by a High Sensitivity Intramolecular FRET Glucose Sensor. Biochim. Biophys. Acta.

[6] Kang Y.P., Ward N.P., DeNicola G.M. (2018). Recent Advances in Cancer Metabolism: A Technological Perspective. Exp. Mol. Med..

[7] Rumsey W., Vanderkooi J., Wilson D. (1988). Imaging of Phosphorescence: A Novel Method for Measuring Oxygen Distribution in Perfused Tissue. Science.

[8] Dmitriev R.I., Papkovsky D.B. (2012). Optical Probes and Techniques for O2 Measurement in Live Cells and Tissue. Cell Mol. Life Sci..

[9] Argüello R.J., Combes A.J., Char R., Gigan J.-P., Baaziz A.I., Bousiquot E., Camosseto V., Samad B., Tsui J., Yan P. (2020). SCENITH: A Flow Cytometry Based Method to Functionally Profile Energy Metabolism with Single Cell Resolution. Cell Metab..

[10] Horan M.P., Pichaud N., Ballard J.W.O. (2012). Review: Quantifying Mitochondrial Dysfunction in Complex Diseases of Aging. J. Gerontol. Ser. A.

[11] Bhagavan N.V., Blanco A., Blanco G. (2017). Chapter 13-Carbohydrate Metabolism I: Glycolysis and TCA Cycle. Medical Biochemistry.

[12] Almeida L., Lochner M., Berod L., Sparwasser T. (2016). Metabolic Pathways in T Cell Activation and Lineage Differentiation. Semin. Immunol..

[13] Bonora M., Patergnani S., Rimessi A., De Marchi E., Suski J.M., Bononi A., Giorgi C., Marchi S., Missiroli S., Poletti F. (2012). ATP Synthesis and Storage. Purinergic Signal..

[14] Rigoulet M., Bouchez C.L., Paumard P., Ransac S., Cuvellier S., Duvezin-Caubet S., Mazat J.P., Devin A. (2020). Cell Energy Metabolism: An Update. Biochim. Biophys. Acta (BBA)-Bioenerg..

[15] Rabinowitz J.D., Enerbäck S. (2020). Lactate: The Ugly Duckling of Energy Metabolism. Nat. Metab..

[16] Lunt S.Y., Vander Heiden M.G. (2011). Aerobic Glycolysis: Meeting the Metabolic Requirements of Cell Proliferation. Annu. Rev. Cell Dev. Biol..

[17] Chang C.-H., Curtis J.D., Maggi L.B., Faubert B., Villarino A.V., O’Sullivan D., Huang S.C.-C., van der Windt G.J.W., Blagih J., Qiu J. (2013). Posttranscriptional Control of T Cell Effector Function by Aerobic Glycolysis. Cell.

[18] Warburg O., Wind F., Negelein E. (1927). The Metabolism of Tumors in the Body. J. Gen. Physiol..

[19] Warburg O. (1956). On the Origin of Cancer Cells. Science.

[20] Liberti M.V., Locasale J.W. (2016). The Warburg Effect: How Does It Benefit Cancer Cells?. Trends Biochem. Sci..

[21] Jones W., Bianchi K. (2015). Aerobic Glycolysis: Beyond Proliferation. Front. Immunol..

[22] Mertens S., Noll T., Spahr R., Krutzfeldt A., Piper H.M. (1990). Energetic Response of Coronary Endothelial Cells to Hypoxia. Am. J. Physiol.-Heart Circ. Physiol..

[23] De Bock K., Georgiadou M., Schoors S., Kuchnio A., Wong B.W., Cantelmo A.R., Quaegebeur A., Ghesquière B., Cauwenberghs S., Eelen G. (2013). Role of PFKFB3-Driven Glycolysis in Vessel Sprouting. Cell.

[24] Lochner M., Berod L., Sparwasser T. (2015). Fatty Acid Metabolism in the Regulation of T Cell Function. Trends Immunol..

[25] Spinelli J.B., Haigis M.C. (2018). The Multifaceted Contributions of Mitochondria to Cellular Metabolism. Nat. Cell Biol..

[26] Yaqoob P., Calder P.C. (1997). Glutamine Requirement of Proliferating T Lymphocytes. Nutrition.

[27] Sinclair L.V., Rolf J., Emslie E., Shi Y.-B., Taylor P.M., Cantrell D.A. (2013). Control of Amino-Acid Transport by Antigen Receptors Coordinates the Metabolic Reprogramming Essential for T Cell Differentiation. Nat. Immunol..

[28] Hope H.C., Salmond R.J. (2021). The Role of Non-Essential Amino Acids in T Cell Function and Anti-Tumour Immunity. Arch. Immunol. Ther. Exp..

[29] Mahnke Y.D., Brodie T.M., Sallusto F., Roederer M., Lugli E. (2013). The Who’s Who of T-Cell Differentiation: Human Memory T-Cell Subsets. Eur. J. Immunol..

[30] Rangel Rivera G.O., Knochelmann H.M., Dwyer C.J., Smith A.S., Wyatt M.M., Rivera-Reyes A.M., Thaxton J.E., Paulos C.M. (2021). Fundamentals of T Cell Metabolism and Strategies to Enhance Cancer Immunotherapy. Front. Immunol..

[31] Saxena A., Dagur P.K., Biancotto A., McCoy J., Philip J. (2019). Multiparametric Flow Cytometry Analysis of Naïve, Memory, and Effector T Cells. Immunophenotyping: Methods and Protocols.

[32] Jacobs S.R., Michalek R.D., Rathmell J.C. (2010). IL-7 Is Essential for Homeostatic Control of T Cell Metabolism In Vivo. J. Immunol..

[33] MacIver N.J., Michalek R.D., Rathmell J.C. (2013). Metabolic Regulation of T Lymphocytes. Annu. Rev. Immunol..

[34] Frauwirth K.A., Riley J.L., Harris M.H., Parry R.V., Rathmell J.C., Plas D.R., Elstrom R.L., June C.H., Thompson C.B. (2002). The CD28 Signaling Pathway Regulates Glucose Metabolism. Immunity.

[35] Pellegrino M., Del Bufalo F., De Angelis B., Quintarelli C., Caruana I., de Billy E. (2020). Manipulating the Metabolism to Improve the Efficacy of CAR T-Cell Immunotherapy. Cells.

[36] Yang K., Shrestha S., Zeng H., Karmaus P.W.F., Neale G., Vogel P., Guertin D.A., Lamb R.F., Chi H. (2013). T Cell Exit from Quiescence and Differentiation into Th2 Cells Depend on Raptor-MTORC1-Mediated Metabolic Reprogramming. Immunity.

[37] Shyer J.A., Flavell R.A., Bailis W. (2020). Metabolic Signaling in T Cells. Cell Res..

[38] Johnson M.O., Wolf M.M., Madden M.Z., Andrejeva G., Sugiura A., Contreras D.C., Maseda D., Liberti M.V., Paz K., Kishton R.J. (2018). Distinct Regulation of Th17 and Th1 Cell Differentiation by Glutaminase-Dependent Metabolism. Cell.

[39] Shi L.Z., Wang R., Huang G., Vogel P., Neale G., Green D.R., Chi H. (2011). HIF1α–Dependent Glycolytic Pathway Orchestrates a Metabolic Checkpoint for the Differentiation of TH17 and Treg Cells. J. Exp. Med..

[40] Michalek R.D., Gerriets V.A., Jacobs S.R., Macintyre A.N., MacIver N.J., Mason E.F., Sullivan S.A., Nichols A.G., Rathmell J.C. (2011). Cutting Edge: Distinct Glycolytic and Lipid Oxidative Metabolic Programs Are Essential for Effector and Regulatory CD4+ T Cell Subsets. J. Immunol..

[41] Wei J., Raynor J., Nguyen T.-L.M., Chi H. (2017). Nutrient and Metabolic Sensing in T Cell Responses. Front. Immunol..

[42] Giraldo N.A., Sanchez-Salas R., Peske J.D., Vano Y., Becht E., Petitprez F., Validire P., Ingels A., Cathelineau X., Fridman W.H. (2019). The Clinical Role of the TME in Solid Cancer. Br. J. Cancer.

[43] Rostamian H., Fallah-Mehrjardi K., Khakpoor-Koosheh M., Pawelek J.M., Hadjati J., Brown C.E., Mirzaei H.R. (2021). A Metabolic Switch to Memory CAR T Cells: Implications for Cancer Treatment. Cancer Lett..

[44] Kishton R.J., Sukumar M., Restifo N.P. (2017). Metabolic Regulation of T Cell Longevity and Function in Tumor Immunotherapy. Cell Metab..

[45] Sterner R.C., Sterner R.M. (2021). CAR-T Cell Therapy: Current Limitations and Potential Strategies. Blood Cancer J..

[46] Weinkove R., George P., Dasyam N., McLellan A.D. (2019). Selecting Costimulatory Domains for Chimeric Antigen Receptors: Functional and Clinical Considerations. Clin. Transl. Immunol..

[47] Zeng H., Cohen S., Guy C., Shrestha S., Neale G., Brown S.A., Cloer C., Kishton R.J., Gao X., Youngblood B. (2016). MTORC1 and MTORC2 Kinase Signaling and Glucose Metabolism Drive Follicular Helper T Cell Differentiation. Immunity.

[48] Guedan S., Chen X., Madar A., Carpenito C., McGettigan S.E., Frigault M.J., Lee J., Posey A.D., Scholler J., Scholler N. (2014). ICOS-Based Chimeric Antigen Receptors Program Bipolar TH17/TH1 Cells. Blood.

[49] Kawalekar O.U., O’Connor R.S., Fraietta J.A., Guo L., McGettigan S.E., Posey A.D., Patel P.R., Guedan S., Scholler J., Keith B. (2016). Distinct Signaling of Coreceptors Regulates Specific Metabolism Pathways and Impacts Memory Development in CAR T Cells. Immunity.

[50] Choi B.K., Lee D.Y., Lee D.G., Kim Y.H., Kim S.-H., Oh H.S., Han C., Kwon B.S. (2017). 4-1BB Signaling Activates Glucose and Fatty Acid Metabolism to Enhance CD8+ T Cell Proliferation. Cell Mol. Immunol..

[51] Teijeira A., Garasa S., Etxeberria I., Gato-Cañas M., Melero I., Delgoffe G.M. (2019). Metabolic Consequences of T-Cell Costimulation in Anticancer Immunity. Cancer Immunol Res..

[52] Li W., Qiu S., Chen J., Jiang S., Chen W., Jiang J., Wang F., Si W., Shu Y., Wei P. (2020). Chimeric Antigen Receptor Designed to Prevent Ubiquitination and Downregulation Showed Durable Antitumor Efficacy. Immunity.

[53] Liu L., Bi E., Ma X., Xiong W., Qian J., Ye L., Su P., Wang Q., Xiao L., Yang M. (2020). Enhanced CAR-T Activity against Established Tumors by Polarizing Human T Cells to Secrete Interleukin-9. Nat. Commun..

[54] Zheng W., O’Hear C.E., Alli R., Basham J.H., Abdelsamed H., Palmer L.E., Jones L.L., Youngblood B., Geiger T.L. (2018). PI3K Orchestration of the in Vivo Persistence of Chimeric Antigen Receptor-Modified T Cells. Leukemia.

[55] Perkins M.R., Grande S., Hamel A., Horton H.M., Garrett T.E., Miller S.M., Latimer H.J., Horvath C.J., Kuczewski M., Friedman K.M. (2015). Manufacturing an Enhanced CAR T Cell Product by Inhibition of the PI3K/Akt Pathway during T Cell Expansion Results in Improved In Vivo Efficacy of Anti-BCMA CAR T Cells. Blood.

[56] Fultang L., Booth S., Yogev O., Martins da Costa B., Tubb V., Panetti S., Stavrou V., Scarpa U., Jankevics A., Lloyd G. (2020). Metabolic Engineering against the Arginine Microenvironment Enhances CAR-T Cell Proliferation and Therapeutic Activity. Blood.

[57] Li W., Zhang L. (2020). Rewiring Mitochondrial Metabolism for CD8+ T Cell Memory Formation and Effective Cancer Immunotherapy. Front. Immunol..

[58] Rostamian H., Khakpoor-Koosheh M., Fallah-Mehrjardi K., Mirzaei H.R., Brown C.E. (2021). Mitochondria as Playmakers of CAR T-Cell Fate and Longevity. Cancer Immunol. Res..

[59] Ferrick D.A., Neilson A., Beeson C. (2008). Advances in Measuring Cellular Bioenergetics Using Extracellular Flux. Drug Discov. Today.

[60] Lardy H.A., Johnson D., McMurray W.C. (1958). Antibiotics as Tools for Metabolic Studies. I. A Survey of Toxic Antibiotics in Respiratory, Phosphorylative and Glycolytic Systems. Arch. Biochem. Biophys..

[61] Öberg K.E. (1961). The Site of the Action of Rotenone in the Respiratory Chain. Exp. Cell Res..

[62] Kim H., Esser L., Hossain M.B., Xia D., Yu C.-A., Rizo J., Van Der Helm D., Deisenhofer J. Structure of Antimycin A1, a Specific Electron Transfer Inhibitor of Ubiquinol−Cytochrome c Oxidoreductase. https://pubs.acs.org/doi/pdf/10.1021/ja990190h.

[63] Baur J.R., Workman M. (1964). Influence of Carbonyl Cyanide Phenylhydrazone Derivatives on the Respiration Rate of Banana Pulp Tissue. Nature.

[64] Wu M., Neilson A., Swift A.L., Moran R., Tamagnine J., Parslow D., Armistead S., Lemire K., Orrell J., Teich J. (2007). Multiparameter Metabolic Analysis Reveals a Close Link between Attenuated Mitochondrial Bioenergetic Function and Enhanced Glycolysis Dependency in Human Tumor Cells. Am. J. Physiol. Cell Physiol..

[65] Liu F., Liu W., Zhou S., Yang C., Tian M., Jia G., Wang H., Zhu B., Feng M., Lu Y. (2020). Identification of FABP5 as an Immunometabolic Marker in Human Hepatocellular Carcinoma. J. Immunother. Cancer.

[66] Köksal H., Dillard P., Juzeniene A., Kvalheim G., Smeland E.B., Myklebust J.H., Inderberg E.M., Wälchli S. (2020). Combinatorial CAR Design Improves Target Restriction. J. Biol. Chem..

[67] Moghimi B., Muthugounder S., Jambon S., Tibbetts R., Hung L., Bassiri H., Hogarty M.D., Barrett D.M., Shimada H., Asgharzadeh S. (2021). Preclinical Assessment of the Efficacy and Specificity of GD2-B7H3 SynNotch CAR-T in Metastatic Neuroblastoma. Nat. Commun..

[68] Lamarche C., Novakovsky G.E., Qi C.N., Weber E.W., Mackall C.L., Levings M.K. (2020). Repeated Stimulation or Tonic-Signaling Chimeric Antigen Receptors Drive Regulatory T Cell Exhaustion. bioRxiv.

[69] Hirabayashi K., Du H., Xu Y., Shou P., Zhou X., Fucá G., Landoni E., Sun C., Chen Y., Savoldo B. (2021). Dual-Targeting CAR-T Cells with Optimal Co-Stimulation and Metabolic Fitness Enhance Antitumor Activity and Prevent Escape in Solid Tumors. Nat. Cancer.

[70] Zhang A., Sun Y., Wang S., Du J., Gao X., Yuan Y., Zhao L., Yang Y., Xu L., Lei Y. (2020). Secretion of Human Soluble Programmed Cell Death Protein 1 by Chimeric Antigen Receptor-Modified T Cells Enhances Anti-Tumor Efficacy. Cytotherapy.

[71] Serganova I., Moroz E., Cohen I., Moroz M., Mane M., Zurita J., Shenker L., Ponomarev V., Blasberg R. (2017). Enhancement of PSMA-Directed CAR Adoptive Immunotherapy by PD-1/PD-L1 Blockade. Mol. Ther. Oncolytics.

[72] Sabatino M., Hu J., Sommariva M., Gautam S., Fellowes V., Hocker J.D., Dougherty S., Qin H., Klebanoff C.A., Fry T.J. (2016). Generation of Clinical-Grade CD19-Specific CAR-Modified CD8+ Memory Stem Cells for the Treatment of Human B-Cell Malignancies. Blood.

[73] Garcia-Canaveras J.C., Heo D., Trefely S., Leferovich J., Xu C., Philipson B.I., Ghassemi S., Milone M.C., Moon E.K., Snyder N.W. (2021). CAR T-Cells Depend on the Coupling of NADH Oxidation with ATP Production. Cells.

[74] Yang Q., Hao J., Chi M., Wang Y., Li J., Huang J., Zhang J., Zhang M., Lu J., Zhou S. (2022). D2HGDH-Mediated D2HG Catabolism Enhances the Anti-Tumor Activities of CAR-T Cells in an Immunosuppressive Microenvironment. Mol. Ther..

[75] Lopaschuk G.D., Wall S.R., Olley P.M., Davies N.J. (1988). Etomoxir, a Carnitine Palmitoyltransferase I Inhibitor, Protects Hearts from Fatty Acid-Induced Ischemic Injury Independent of Changes in Long Chain Acylcarnitine. Circ. Res..

